# Exploring the Link between Germline and Somatic Genetic Alterations in Breast Carcinogenesis

**DOI:** 10.1371/journal.pone.0014078

**Published:** 2010-11-22

**Authors:** Núria Bonifaci, Bohdan Górski, Bartlomiej Masojć, Dominika Wokołorczyk, Anna Jakubowska, Tadeusz Dębniak, Antoni Berenguer, Jordi Serra Musach, Joan Brunet, Joaquín Dopazo, Steven A. Narod, Jan Lubiński, Conxi Lázaro, Cezary Cybulski, Miguel Angel Pujana

**Affiliations:** 1 Biomarkers and Susceptibility Unit, Spanish Biomedical Research Centre Network for Epidemiology and Public Health, Catalan Institute of Oncology, L'Institut d'Investigació Biomèdica de Bellvitge (IDIBELL), L'Hospitalet, Barcelona, Spain; 2 Department of Genetics and Pathology, International Hereditary Cancer Center, Pomeranian Medical University, Szczecin, Poland; 3 Hereditary Cancer Programme, Catalan Institute of Oncology, IdIBGi, Girona, Spain; 4 Department of Bioinformatics and Genomics, Centro de Investigación Príncipe Felipe, Functional Genomics Node and Spanish Biomedical Research Centre Network for Rare Diseases, Valencia, Spain; 5 Womens College Research Institute, University of Toronto and Women's College Hospital, Toronto, Ontario, Canada; 6 Hereditary Cancer Programme, Catalan Institute of Oncology, IDIBELL, L'Hospitalet, Barcelona, Spain; 7 Translational Research Laboratory, Catalan Institute of Oncology, IDIBELL, L'Hospitalet, Barcelona, Spain; The University of Hong Kong, China

## Abstract

Recent genome-wide association studies (GWASs) have identified candidate genes contributing to cancer risk through low-penetrance mutations. Many of these genes were unexpected and, intriguingly, included well-known players in carcinogenesis at the somatic level. To assess the hypothesis of a germline-somatic link in carcinogenesis, we evaluated the distribution of somatic gene labels within the ordered results of a breast cancer risk GWAS. This analysis suggested frequent influence on risk of genetic variation in loci encoding for “driver kinases” (i.e., kinases encoded by genes that showed higher somatic mutation rates than expected by chance and, therefore, whose deregulation may contribute to cancer development and/or progression). Assessment of these predictions using a population-based case-control study in Poland replicated the association for rs3732568 in *EPHB1* (odds ratio (OR) = 0.79; 95% confidence interval (CI): 0.63–0.98; *P_trend_* = 0.031). Analyses by early age at diagnosis and by estrogen receptor α (ERα) tumor status indicated potential associations for rs6852678 in *CDKL2* (OR = 0.32, 95% CI: 0.10–1.00; *P_recessive_* = 0.044) and rs10878640 in *DYRK2* (OR = 2.39, 95% CI: 1.32–4.30; *P_dominant_* = 0.003), and for rs12765929, rs9836340, rs4707795 in *BMPR1A*, *EPHA3* and *EPHA7*, respectively (ERα tumor status *P_interaction_*<0.05). The identification of three novel candidates as *EPH receptor* genes might indicate a link between perturbed compartmentalization of early neoplastic lesions and breast cancer risk and progression. Together, these data may lay the foundations for replication in additional populations and could potentially increase our knowledge of the underlying molecular mechanisms of breast carcinogenesis.

## Introduction

With the advent of technical and methodological advances, several GWASs identifying common genetic variation associated with risk of developing cancer have been completed recently [1]. Thus, initiatives such as the National Cancer Institute's Cancer Genetic Markers of Susceptibility (CGEMS) and efforts carried out by deCODE Genetics and the Breast Cancer Association Consortium have led to the identification of breast cancer risk alleles in single nucleotide polymorphisms (SNPs) replicated across populations [2]–[6]. Intriguingly, illustrating the unbiased nature of GWASs, most hits have corresponded to *a priori* unexpected candidate genes. In this context, the involvement of biological processes beyond the canonical DNA damage response in breast cancer is further suggested by the observed differential influence of low-penetrance risk alleles among *BRCA1* and *BRCA2* mutation carriers [7]–[9].

A potential common characteristic of the unexpected low-penetrance susceptibility genes is the previously identified contribution to tumorigenesis, but at the somatic level. Common genetic variation in loci encoding for *FGFR2* and *MAP3K1* influences risk of breast cancer [2], [4], and these genes were previously found to be somatically mutated in diverse neoplasias including breast cancer [10], [11]. In addition, and central to the understanding of cancer progression, common risk alleles showed differential influence according to ERα tumor status [12], and variation in the locus encoding for ERα, *ESR1*, also influences risk of breast cancer [13], [14]. More recently, additional breast cancer susceptibility loci have been described that include *CDKN2A/B* as candidates [15]. While these observations suggest a “germline-somatic” link in breast carcinogenesis, an analogous situation may exist for other neoplasias. Variation in loci encoding for *CDH1* and *SMAD7* influences risk of colorectal cancer [16], [17] and, similarly, these genes were previously identified as inactivated or deregulated in tumors [18]–[21]. Moreover, deregulated germline expression of a paradigmatic proto-oncogene, *MYC*, may be a common mechanism of tumorigenesis in epithelial tissues [22]–[25]. However, despite some evidence of a germline-somatic link, as yet there is no explicit evaluation of this hypothesis and its potential usefulness in replication studies. Here we present an examination of this link through analysis of the CGEMS GWAS breast cancer dataset and subsequent assessment of the predictions in a case-control study of incident breast cancer in Poland.

## Results

### Distribution of somatic gene sets in ordered breast cancer GWAS results

**Figure 1:**
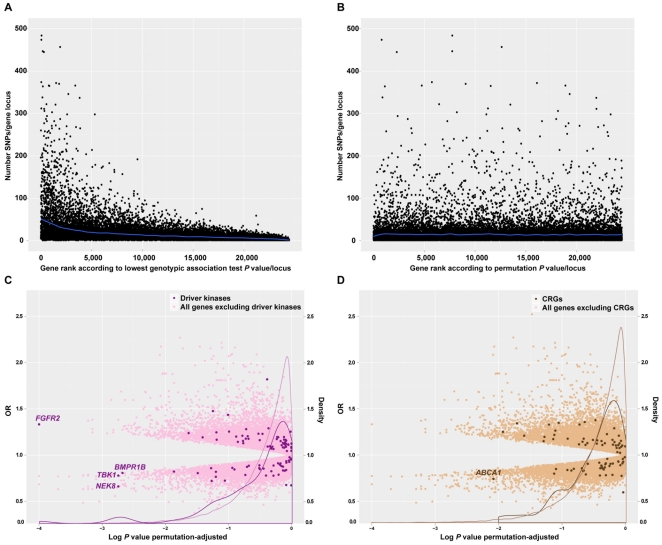
GWAS ranks and distribution of cancer somatic gene sets. *A*, Original GWAS results ranked according to the lowest genotypic association test *P* value per gene locus (unadjusted for genomic extension; taken SNPs in defined genomic window of ±10 kb relative to the first and last exons of a given gene). The Y-axis indicates the number of SNPs per gene locus while the X-axis indicates the lowest association *P* value per gene locus. Bias can be appreciated as the number of SNPs per gene locus increases at lower *P* values. *B*, GWAS results ranked according to the lowest association *P* value per gene locus but adjusted by genomic extension through case-control permutations. Compared to the previous graph, the bias largely disappears. *C*, Following the rank in *B*, the Y-axis indicates odds ratios (ORs) of allele effects and density distributions of gene sets (driver kinases correspond to a light lilac curve; the rest of the genome in the GWAS dataset is shown by a dark lilac curve), while the X-axis indicates the log-transformed association *P* values, previously adjusted by genomic extension. As indicated by the density curves, SNPs mapping to driver kinase loci are relatively more frequent at lower association adjusted *P* values. This observation is supported by GSEA results using the same CGEMS GWAS adjusted rank; nominal *P*<0.001 and FDR-adjusted *P* = 0.010 (Table S2). *D*, Similarly to the graph in *C*, distribution of CRGs in the CGEMS GWAS rank adjusted through permutations.

Previously, analysis of the CGEMS GWAS dataset using the lowest genotypic *P* value per gene locus suggested true associations in genes annotated with Gene Ontology (GO) biological process terms linked to somatic events [26], [27]. However, since there is a positive correlation between the extension of a given locus and the number of SNPs it may contain (and, therefore, the possibility of significant association results being obtained by chance), an unadjusted GWAS rank is biased at its lowest *P* values for specific processes in which large gene products frequently participate [26], [28], [29] (Fig. 1*A*). Nevertheless, cancer genes tend to expand across large genomic regions [30], and examination of eight genes likely involved in breast cancer through low-penetrance mutations–*CASP8*, *COX11*, *ESR1*, *FGFR2*, *LSP1*, *MAP3K1*, *RAD51L1* and *TOX3* [2]–[6], [13], [14]–showed a trend for larger genomic loci (mean (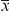) genomic extension = 211 kilo bases (kb) and standard deviation (σ) = 283 kb; compared to 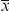 = 66 kb and σ = 128 kb for all annotated genes in the CGEMS GWAS rank).

Having identified caveats to the ranking of GWAS results, we performed 10,000 permutations of case-control status and used the null distribution of *t* statistics from the age-adjusted partial correlation analysis to correct the original rank, which then showed an unbiased distribution (Fig. 1*B*). Prior to the evaluation of somatic sets, analysis of GO biological process terms in the GWAS permutation *P* values rank did not show any significant asymmetry using the Gene Set Enrichment Analysis (GSEA) tool [31] with multiple testing correction by the false discovery rate (FDR) approach [32]. Nonetheless, most processes with nominally significant *P* values were those previously highlighted, which are associated with somatic events [26], [27] (Table S1). This observation appears to agree with recently described results of pathway-based analysis of the same GWAS dataset [33].

Next, evaluation of somatic sets related to cancer prognosis and treatment response prediction, and to genetic and genomic alterations (see Materials and Methods), revealed significant asymmetrical distribution of “driver kinases” [34], [35]; that is, kinases whose deregulation through frequent somatic mutation contributes to tumor development and/or progression (“driver mutations”). In contrast, “passenger mutations” were defined as essentially neutral and linked to the inherent genetic instability in cancer cells [34], [35]. Thus, the driver kinases set was found to be biased towards the top (nominal significant association results) of the GWAS permutation rank (GSEA nominal *P*<0.001; FDR-adjusted *P* = 0.010) (Fig. 1*C* and Table S2). Among the remaining of somatic sets evaluated, only cooperation response genes (CRGs) to oncogenic mutations [36] showed a trend for a distribution similar to that of driver kinases (GSEA nominal *P* = 0.080; FDR-adjusted *P* value = 0.25) (Fig. 1*D*), although the intersection between both sets only contained two genes (Table S2). Therefore, in somatic cancer genes, common genetic variation in driver kinase loci might frequently influence risk of breast cancer.

The set of driver kinases contained a benchmark gene, *FGFR2* [2], [4], and a locus recently replicated in an independent study, *BMPR1B* [37]. Nevertheless, a significant bias was still observed following exclusion of these two loci (GSEA nominal *P* = 0.001; FDR-adjusted *P* = 0.048), which suggests that variation at additional driver kinase loci influences risk of breast cancer. Importantly, using the set of non-driver kinases–either the subsequent equivalent set as originally statistically ordered or the total set (*n* = 344) [35]–did not reveal significant bias (GSEA nominal *P* = 0.99 and 0.66, respectively), which reinforces the idea of frequent involvement of driver kinases. However, if only the individual statistical data for each locus were considered, most of the driver kinase loci would perhaps not have been selected for replication in other populations.

### Independent association results for common variation in driver kinase loci

Given the possible bias in GWAS rank identified above, we examined the top 20 driver kinase variants in the original rank (Table S3, including details of the CGEMS and results below) in a case-control study of incident breast cancer in Szczecin (Poland), previously used in other replications [38]. Applying genotyping quality controls and Hardy-Weinberg equilibrium analysis, 16 SNPs representing an identical number of driver kinase loci (i.e., a single SNP for each locus and representing the strongest potential statistical association) were examined for their association with risk of breast cancer using 880 controls and 1,173 cases (see Materials and Methods). In this analysis, the rs3732568 variant in the *ephrin type-B receptor 1* (*EPHB1*) locus was found to be associated with risk of breast cancer: OR = 0.79, 95% CI: 0.63–0.98; *P_trend_* = 0.031 (Table 1). Further evaluation of this association through 10,000 case-control permutations in our study gave a similar significance value, *P_trend_* = 0.034. Importantly, this association was in the same direction and with similar magnitude to the result in the CGEMS GWAS: age-adjusted OR = 0.78, 95% CI: 0.64–0.94; *P_trend_* = 0.009.

**Table 1:** Association between genetic variation in *EPHB1* and risk of breast cancer in Poland.

*EPHB1*, rs3732568						
	Controls		Cases			
	*n*	%	*n*	%	OR	95% CI
C/C	693	79.8	891	83.2	1.00	
C/A	165	19.0	172	16.1	0.79	0.62–1.00
A/A	10	1.2	8	0.7	0.60	0.23–1.55
Total	868		1,071			
Trend					0.79	0.63–0.98
					*P_trend_* = 0.031†	

† Adjusted by age.

**Figure 2:**
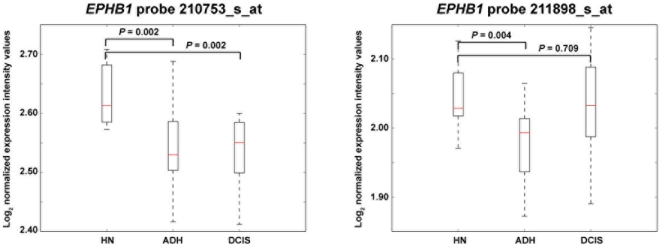
Early change of *EPHB1* expression in breast carcinogenesis. The graphs show expression profiles in histologically normal (HN) breast tissues versus patient-matched atypical ductal hyperplasia (ADH) and ductal carcinoma *in situ* (DCIS) [41]. Results of two *EPHB1* microarray probes (names shown at the top) and the corresponding significance *P* values are shown.

While deregulated expression or function of EPHs and EPH receptors is thought to play a critical role in the initial stages of epithelial neoplasia [39], [40], recent analysis of early breast cancer expression changes suggests a link between disruption of cell adhesion and extracellular matrix pathways, and the risk of developing breast cancer [41]. Analysis of this recent dataset also revealed an early expression change of *EPHB1*, between normal breast tissue and atypical ductal hyperplasia (Fig. 2). This alteration consisted of infra-expression in hyperplasia, akin to its potential role in the compartmentalization of early neoplastic lesions [42]. Together, association studies, early expression changes in carcinogenesis and the regulation of cell adhesion suggest the involvement of *EPHB1* in risk of breast cancer.

Next, given accepted models of inherited breast cancer susceptibility [43], we examined associations with risk at early age of diagnosis (≤40 years old). This analysis indicated two additional potential associations: rs6852678 in *CDKL2*, recessive model OR = 0.32, 95% CI: 0.10–1.00; *P* = 0.044; and rs10878640 in *DYRK2*, dominant model OR = 2.39, 95% CI: 1.32–4.30; *P* = 0.003 (Table 2). Results for rs6852678 appeared to be consistent with CGEMS GWAS analysis; age-adjusted recessive model OR = 0.71, 95% CI: 0.53–0.95; *P* = 0.019; however, the pattern for rs10878640 might be more complex (CGEMS GWAS ORs = 1.05 and 0.68 for heterozygotes and minor allele homozygotes, respectively).

**Table 2:** Associations between genetic variation in driver kinase loci and risk of breast cancer at ≤40 years of first age at diagnosis.

*CDKL2*, rs6852678						
	Controls		Cases			
	*n*	%	*n*	%	OR	95% CI
C/C	39	51.3	62	51.2	1.00	
C/T	28	36.8	54	44.6	1.21	0.66–2.23
T/T	9	11.8	5	4.1	0.35	0.11–1.12
Total	76		121			
Recessive					0.32	0.10–1.00
					*P_recessive_* = 0.044	

*DYRK2*, rs10878640						
	Controls		Cases			
	*n*	%	*n*	%	OR	95% CI
G/G	42	56.8	44	35.5	1.00	
G/T	24	32.4	66	53.2	2.62	1.40–4.93
T/T	8	10.8	14	11.3	1.67	0.64–4.39
Total	74		124			
Dominant					2.39	1.32–4.30
					*P_dominant_* = 0.003	

Having potential differences by ERα tumor status, we next examined associations in ERα-positive and -negative breast cancer patients. Thus, rs3732568 in *EPHB1* showed a similar influence on either type of breast cancer (Table 3)–which is consistent with an overall significant association–and rs12765929 in *BMPR1A* and rs9836340 in *EPHA3* showed a potential major impact on the risk of ERα-negative breast cancer (*P* for difference in OR (interaction) by ERα status <0.05), while rs4707795 in *EPHA7* showed a differential effect between ERα-negative versus ERα-positive breast cancer risk (*P_interaction_* = 0.007) (Table 3). None of these additional candidates linked to ERα tumor status, or those linked to an early age of diagnosis above, showed significant expression differences at early stages of breast carcinogenesis as *EPHB1*. On the other hand, the remaining SNPs examined in this study after applying quality controls and Hardy-Weinberg equilibrium analysis (i.e., 10 out of 16), did not show significant associations following CGEMS evidence (Table S3). Together, the gene-set based analysis of GWAS data and the subsequent replication attempt might indicate that common genetic variation in specific driver kinase loci, and particularly in *EPH receptor* genes, influence risk of breast cancer.

**Table 3:** Associations of genetic variation in driver kinase loci and risk of breast cancer by ERα tumor status†.

*BMPR1A*, rs12765929										
	Controls		ERα-negative				ERα-positive			
	*n*	%	*n*	%	OR	95% CI	*n*	%	OR	95% CI
G/G	514	59.1	189	64.5	1.00		389	58.4	1.00	
G/T	306	35.2	96	32.8	0.87	0.65–1.16	243	36.5	1.07	0.86–1.33
T/T	50	5.7	8	2.7	0.45	0.21–0.98	34	5.1	0.93	0.59–1.48
Total	870		293				666			
Trend					0.79	0.62–1.00			1.02	0.86–1.21
					*P_trend_* = 0.050				*P_trend_* = 0.81*P_interaction_* = 0.024	

*EPHB1*, rs3732568										
	Controls		ERα-negative				ERα-positive			
	*n*	%	*n*	%	OR	95% CI	*n*	%	OR	95% CI
C/C	693	79.8	242	82.6	1.00		563	84.9	1.00	
C/A	165	19.0	49	16.7	0.81	0.57–1.16	94	14.2	0.68	0.51–0.90
A/A	10	1.2	2	0.7	0.55	0.12–2.56	6	0.9	0.72	0.26–2.00
Total	868		293				663			
Trend					0.80	0.58–1.11			0.71	0.55–0.91
					*P_trend_* = 0.18				*P_trend_* = 0.007*P_interaction_* = 0.56	

*EPHA3*, rs9836340										
	Controls		ERα-negative				ERα-positive			
	*n*	%	*n*	%	OR	95% CI	*n*	%	OR	95% CI
A/A	446	51.3	154	52.4	1.00		356	53.7	1.00	
A/G	341	39.2	99	33.7	0.84	0.63–1.13	251	37.9	0.91	0.74–1.14
G/G	82	9.5	41	13.9	1.43	0.93–2.19	56	8.4	0.85	0.58–1.22
Total	869		294				663			
Recessive					1.53	1.02–2.31			0.88	0.61–1.26
					*P_recessive_* = 0.040				*P_recessive_* = 0.48*P_interaction_* = 0.010	

*EPHA7*, rs4707795										
	Controls		ERα-negative				ERα-positive			
	*n*	%	*n*	%	OR	95% CI	*n*	%	OR	95% CI
G/G	618	71.0	204	69.6	1.00		479	71.9	1.00	
G/A	239	27.5	87	29.7	1.18	0.88–1.60	166	24.9	0.92	0.73–1.17
A/A	13	1.5	2	0.7	0.45	0.10–2.06	21	3.2	2.11	1.04–4.28
Total	870		293				666			
Recessive					0.43	0.10–1.96			2.15	1.06–4.37
					*P_recessive_* = 0.28				*P_recessive_* = 0.034*P_interaction_* = 0.007	

† Adjusted by age.

## Discussion

Evaluation of a germline-somatic link in breast carcinogenesis suggests a role for driver kinases and, perhaps to a lesser extent, genes with a synergistic response to oncogenic mutations. This study might be limited by the assignment of the lowest genotypic *P* value per gene locus within a defined genomic window (i.e., ±10 kb)–thus excluding a large proportion of variation that cannot be assigned to a specific known gene–and by its focus on the additive model of influence of risk alleles when adjusted through case-control permutations. Future analyses taking into account the potential perturbation of germline gene expression by, for example, common variation at distant regulatory regions may improve the identification of susceptibility genes using GWAS complete data. Another limitation in the interpretation of the results presented here may lie in the case-control study designs: the CGEMS addressed breast cancer risk in postmenopausal women, while the Polish study was relatively enriched in early-onset cases. Therefore, studies in additional populations, with diverse designs, are warranted to corroborate the results shown here.

The results of the replication study may be consistent with previously detected somatic genetic alterations and/or functional roles. Somatic mutations in *CDKL2* were nonsense and were only detected in breast and ovarian cancer cell lines or tumors [11], [35]. CDKL2 (also known as p56 or KKIAMRE) is the most distant member of the CDC2-related serine/threonine protein kinase family, involved in epidermal growth factor signaling [44], but with a mostly uncharacterized function. *DYRK2* was found to be mutated in breast and central nervous system tumors, in nonsense and missense alterations, respectively [11], [35]. The functional role of DYRK2 in the DNA damage response [45] may link to CGEMS GWAS results for *RAD51L1* [3]: loss of DYRK2 function alters the activation of apoptosis in response to DNA damage via ATM [45], which may therefore promote carcinogenesis.

Having revealed potential associations linked to known somatic alterations, the most striking results of this study may concern the identification of risk alleles at three *EPH receptor* loci. EPH-mediated signaling regulates important biological process altered in carcinogenesis, such as cell-to-cell communication, and cell migration and adhesion via the actin cytoskeleton [39], [40]. Thus, through RHO and RAS/MAPK activities [46], this signaling pathway has been implicated in the maintenance of epithelial tissue architectures and is therefore thought to act as a tumor suppressor [39], [40]. These observations may indicate that, similarly to colorectal tumorigenesis [42], EPH-mediated compartmentalization of early breast tissue neoplastic lesions is critical to prevent the subsequent emergence of carcinoma. Therefore, through a germline expression or functional perturbation, *EPHB1* may contribute to the observed variability in the transition from an *in situ* lesion to an invasive carcinoma [47]. While the associations revealed here warrant further replication in other populations, the existing data could potentially increase current knowledge of the genetic basis and molecular mechanisms of breast carcinogenesis.

## Materials and Methods

### CGEMS dataset

The National Cancer Institute CGEMS initiative has conducted genome-wide association studies to identify common genetic variants and the corresponding functionally affected genes involved in breast cancer and prostate cancer susceptibility. An initial CGEMS whole genome scan was designed to study the main effect of SNPs on breast cancer risk in postmenopausal women [2]. The study involved 1,145 invasive postmenopausal breast cancer cases and 1,142 matched controls from the Nurses' Health Study nested case-control study [48]. Results of the CGEMS GWAS of breast cancer were obtained upon approval of a Data Access Request.

### GWAS rank

In our previous analyses [26], [27], ordered CGEMS GWAS results (i.e., ranks) corresponded to the lowest *P* value per gene for the genotypic test in a genomic region of +/−10 kb at each gene locus, defined by the Ensembl human genome release 57. Assigned SNPs were curated using Ensembl gene annotations. We [26] and others [28] noted that such ranks were biased along with the genomic extension–and therefore with the number of SNPs–per gene locus. To adjust for this bias, several statistical strategies are possible [28], including carrying out permutations of the case-control status to correct the significance of the original statistic. In our analysis, considering typed and informative SNPs in each gene locus, we first chose the maximum absolute value of the *t* statistic from the age-adjusted partial correlation in the additive model. Next, 10,000 permutations of the same informative SNPs were performed to create a null distribution for this maximum *t* statistic, which was used to assess its significance corrected by number of SNPs.

### GSEA application

The distribution of gene sets in ranked GWAS results was examined using the non-parametric algorithm in the GSEA tool, with default values for all parameters [31] except for the set size when appropriated. In GSEA, a pre-defined gene set is mapped to a rank–in our case genes/loci ordered according to the adjusted association statistic–to assess potential bias using an enrichment score that reflects the degree to which this set is overrepresented at the extremes of the entire ranked list. In the interpretation of the results, caution should be taken when considering sets of different size. In our study, different hypotheses were examined independently (i.e., gene sets linked to prognosis, prediction or genetic/genomic somatic alterations), and *P* values were corrected for multiple testing within each group : 1) genes whose expression in primary breast tumors was associated with patient prognosis and/or metastasis [49]–[55]; 2) genes whose expression in primary breast tumors was associated with patient therapeutic treatment response [56]–[59]; 3) genes whose expression levels differed according to ERα breast tumor status or grade [60], or in response to 17β-estradiol [61]; and 4) genes with somatic genetic and/or genomic somatic alterations (Table S2). This last group was made up of five sets : i/ driver kinases (conditional probability of containing driver mutations >0.70, *n* = 119 as defined previously [35], of which 95 were uniquely mapped in the GWAS rank); ii/ CRGs to oncogenic mutations [36]; iii/ cancer gene census, somatically-mutated only [62], [63]; iv/ genes affected by somatic chromosomal rearrangements and/or fusions [64]; and v/ amplified and over-expressed cancer genes [65] (Table S2).

### Gene expression analysis

Raw expression microarray data on breast cancer progression [41] were downloaded from the Gene Expression Omnibus reference GSE16873 and normalized with robust multiarray average (RMA) [66] and significance analysis was performed using the significance analysis of microarray (SAM) algorithm [67].

### Study samples in Poland and association study

A case-control study of unselected invasive breast cancer collected between 1996 and 2003 in Szczecin (Poland) was analyzed. The series included 976 cases of breast cancer unselected for age and an additional group of 367 cases of breast cancer diagnosed at age 50 or below. Therefore, the series was enriched for early-onset cases: mean age of diagnosis was 52.4 years (range 19–88). Subjects were unselected for family history and 15% of cases reported a first- or second-degree relative with breast cancer. The participation rate exceeded 70% among women with breast cancer invited to enroll. Collected information included year of birth, age at diagnosis of breast and/or ovarian cancer, tumor bilaterality, family history (first- and second-degree relatives with breast and/or ovarian cancer) and tumor pathological features in >80% of cases (ERα and progesterone receptor status, and grade). Cases were also examined for *BRCA1* founder mutations in Poland [68] and, if positive, excluded from the association study (*n* = 50). The control group included cancer-free adult women from the same population (920 women with mean age of diagnosis of 56.7, range 20–91) taken from the healthy adult patients of five family doctors practicing in the Szczecin region. These individuals were selected randomly from the patient lists of the participating doctors. The study was carried out with informed consent of the probands and approved by local ethics committees. Genotypes were obtained using Sequenom iPLEX chemistry at the International Hereditary Cancer Center. Quality controls were of >95% calling for each SNP and >90% of calls per sample. Thus, in the set of 16 SNPs, we observed an average concordance rate of 98.7% of genotype calls using 3.3% replicates. Genotypes of 880 controls and 1,173 cases were effectively analyzed using conditional and unconditional logistic regressions (age adjustment using similar strata size; 20–46, 46–56, 56–66, and 66–91 years old).

## Supporting Information

Table S1 (0.02 MB XLS)

Table S2 (0.05 MB XLS)

Table S3 (0.03 MB XLS)
